# Editorial: Advances in research on the role of dna damage repair in reproductive diseases

**DOI:** 10.3389/fendo.2023.1357497

**Published:** 2024-01-08

**Authors:** Kai Meng, Mengmeng Yao, Chuqi Liu, Lei Yang

**Affiliations:** ^1^ Lin He’s Academician Workstation of New Medicine and Clinical Translation, Jining Medical University, Jining, China; ^2^ College of Second Clinical Medical, Jining Medical University, Jining, China; ^3^ Wenzhou Institute, University of Chinese Academy of Sciences, Wenzhou, Zhejiang, China

**Keywords:** DNA damage, DNA repair, reproductive diseases, human infertility, reproduction

During the intricate processes of replication and expression, DNA is susceptible to damage from various exogenous and endogenous factors. Such damage can impact the formation of germ cells and may even contribute to the development of infertility in humans. The accurate repair of DNA damage plays a pivotal role in maintaining the stability of the germ cell genome. Nevertheless, a comprehensive understanding and refinement of the DNA repair mechanism by humans remain elusive. Therefore, we are pleased to establish a Research Topic consisting of four articles (Wang et al., Akhigbe et al., Ratnayaka-Gamage et al., Jiang et al.) to systematically explore the causes of DNA damage associated with germ cell genesis and development and its repair mechanisms in order to provide new clues for the treatment of human infertility diseases.

In the process of exploring the causes of DNA damage, Wang et al. comprehensively and systematically outlined endogenous DNA damage factors such as replication errors, spontaneous chemical changes and base reciprocal isomerism, as well as exogenous DNA damage factors such as physical rays such as x-rays and ultraviolet irradiation, chemical reagents such as methyl thiomethane, and biological factors such as aflatoxins, among others, in a large number of literatures. In addition, Akhigbe et al. uncovered that both maternal and prepubertal codeine exposure induced oxidative DNA damage in sperm and reduced mRNA levels encoding spermatogenic genes, thereby impairing spermatogenesis in F1 males. Jiang et al. suggested that obesity may increase oocyte DNA damage and affect the ability of oocytes to repair sperm DNA damage, thereby affecting embryo development and reproductive outcomes. In exploring DNA repair pathways, Wang et al. outlined four pathways for DNA excision and recombination repair: base excision repair (BER), nucleotide excision repair (NER), homologous recombination repair (HRR), and nonhomologous recombination repair (HRD). Ratnayaka-Gamage et al. proposed that KU80 (encoded by the XRCC5 gene) is not essential for DNA double-strand break (DSB) repair in primordial follicular oocytes, that KU80 deletion in oocytes does not affect ovarian reserve or follicular atresia, and provided support for the hypothesis that the homologous recombination (HR) repair pathway is the primary pathway for DSB repair in follicular oocytes, by subjecting oocyte specific Xrcc5 conditional knockout (Xrcc5 cKO) mice to a control treatment with wildtype (WT) mice. Finally, Wang et al. provided an overview of commonly used drugs, such as icariin, antioxidants, and poly (ADP-ribose) polymerase (PARP) inhibitors, in the treatment of DNA damage and reproductive disorders. They also discussed treatments such as intracytoplasmic sperm injection (ICSI) and varicocele surgery. In addition to the discovery of codeine’s ability to damage DNA, Akhigbe et al. found that pre-pubertal arginine supplementation attenuated the negative effects of codeine on male reproduction. However, further experiments are needed to elucidate whether oxidative DNA damage is reversed or if DNA damage is repaired through this supplementation.

In conclusion, the articles within this topic emphasize the substantial and detrimental influence of DNA damage on reproduction. They underscore the pivotal role of precise DNA repair in the complex processes of germ cell genesis and development, underscoring its significance in the treatment of reproductive diseases. Our hope is that this Research Topic will establish itself as a fundamental resource for future inquiries in this field, providing novel insights that could contribute to the progress of treatments for human infertility.

## Author contributions

KM: Writing – original draft, Writing – review & editing. MY: Writing – original draft. CL: Writing – original draft. LY: Writing – review & editing.

